# Exploring the Inflammatory Basis of Endometrial Polyps: Clinical Implications of Hematological Biomarkers in a Retrospective Study

**DOI:** 10.3390/jcm14082754

**Published:** 2025-04-17

**Authors:** Betül Keyif, Ali Yavuzcan, Engin Yurtçu, Alper Başbuğ, Fatmanur Düzenli, Elif Keyif, Fikret Gokhan Goynumer

**Affiliations:** 1 Department of Obstetrics and Gynecology, Faculty of Medicine, Duzce University, 81620 Duzce, Türkiye; drenginyurtcu1@hotmail.com (E.Y.); dralper23@gmail.com (A.B.); fatmanur_duzenli@hotmail.com (F.D.); goynumergo@gmail.com (F.G.G.); 2 Department of Obstetrics and Gynecology, University of Health Sciences Ankara City Health Application and Research Centre, 06350 Ankara, Türkiye; draliyavuzcan@yahoo.com; 3 Department of Obstetrics and Gynecology, Bolu Izzet Baysal State Hospital, 14300 Bolu, Türkiye; satirogluelif@gmail.com

**Keywords:** chronic endometritis, endometrial polyp, hematological biomarkers, inflammation, mean platelet volume, neutrophil-to-lymphocyte ratio, platelet-to-lymphocyte ratio

## Abstract

**Background/Objectives**: Endometrial polyps (EPs) are common benign endometrial lesions often linked to abnormal uterine bleeding and infertility. While hormonal factors play a key role in their development, recent studies suggest a potential inflammatory component. This retrospective study aimed to assess systemic inflammatory markers, including mean platelet volume (MPV), neutrophil-to-lymphocyte ratio (NLR), and platelet-to-lymphocyte ratio (PLR), in EP patients. **Methods**: A total of 180 patients were classified into three groups: EP (n = 60), chronic endometritis (n = 60), and control (n = 60). Preoperative hematological parameters were retrieved from medical records. Group comparisons were performed using one-way ANOVA, with Tukey’s post hoc test applied when significant. Multinomial logistic regression was used to identify independent predictors of EPs. **Results**: MPV and PLR were significantly higher in the EP group compared to other groups (*p* = 0.014 and *p* = 0.015, respectively), while NLR differences were not statistically significant (*p* = 0.086). Logistic regression identified MPV (*p* = 0.004) and PLR (*p* = 0.045) as independent predictors of EPs. **Conclusions**: These findings suggest that systemic inflammation may contribute to EP development, with MPV and PLR serving as potential inflammatory biomarkers. Further prospective studies with histopathological validation are needed to clarify the role of inflammation in EP pathogenesis.

## 1. Introduction

Endometrial polyps (EPs) are focal overgrowths of the endometrial mucosa, frequently associated with abnormal uterine bleeding (AUB) and infertility. The prevalence of EPs varies between 10% and 40% among reproductive-aged and postmenopausal women. While most EPs exhibit benign characteristics, some may harbor atypical hyperplasia or malignant transformation [1,2]. Traditionally, EPs have been regarded as hormonally driven lesions, primarily influenced by estrogen and progesterone imbalances. However, emerging evidence suggests that chronic inflammation may play a crucial role in their pathogenesis [3].

Chronic endometritis (CE) is a persistent inflammatory condition of the endometrium, characterized by the presence of CD-138-positive plasma cells in the endometrial stroma and glandular epithelium [4]. A recent meta-analysis reported that the prevalence of CE in women with EPs is 51.35%, with EPs being three times more likely to occur in women with CE compared to those without CE [5]. This finding underscores the hypothesis that EPs may not solely be a hormonal disorder but rather a condition influenced by chronic inflammatory processes.

Systemic inflammatory markers such as neutrophil-to-lymphocyte ratio (NLR), platelet-to-lymphocyte ratio (PLR), and mean platelet volume (MPV) have been widely investigated as indicators of systemic inflammation in various pathological conditions, including cardiovascular diseases, malignancies, and autoimmune disorders [6,7]. Despite their well-established role in systemic inflammation, the potential significance of these hematological biomarkers in EP pathogenesis remains unexplored. It has been postulated that NLR and PLR could serve as markers of systemic inflammatory activity, while MPV may reflect platelet activation and the vascular component of inflammation [8,9].

Although prior studies have demonstrated an association between EPs and CE, the relationship between EPs and systemic inflammatory markers remains largely unknown [10,11]. This study aims to address this gap by evaluating the levels of NLR, PLR, and MPV in patients with EPs and exploring their potential role in the inflammatory pathogenesis of these lesions. Our study is among the first to investigate whether these hematologic markers could serve as potential biomarkers for EP development, offering new insights into the interplay between systemic inflammation and localized endometrial pathology.

This study aims to assess MPV, NLR, and PLR levels in women with endometrial polyps and determine their potential role in EP pathogenesis. To achieve this, we will compare these hematological parameters among three groups: patients with EPs, patients with endometritis, and healthy individuals with normal endometrial histology. By elucidating the role of systemic inflammation in EP development, our findings may provide a novel perspective on the inflammatory mechanisms underlying endometrial pathology.

## 2. Materials and Methods

### 2.1. Study Design and Ethical Considerations

This retrospective, case–control study was conducted at the Department of Obstetrics and Gynecology, Düzce University Faculty of Medicine, between February 2022 and April 2022. The study was performed in accordance with the ethical principles outlined in the Helsinki Declaration, and ethical approval was obtained from the Düzce University Clinical Research Ethics Committee (Approval No.: 2022/55, Date: 25 April 2022). Due to the retrospective nature of the study, written informed consent was waived by the ethics committee. Patient confidentiality was strictly maintained, and all data were anonymized before analysis.

### 2.2. Study Population and Groups

The study included female patients who underwent hysteroscopic evaluation, polypectomy, or diagnostic endoscopic procedures. Based on clinical and hematological findings retrieved from medical records, the study population was classified into three groups. The endometrial polyp group consisted of patients diagnosed with endometrial polyps based on hysteroscopic findings, which revealed sessile or pedunculated endometrial overgrowths consistent with polyp formation. The endometritis group included patients with a clinical diagnosis of chronic endometritis, determined by clinical history, hysteroscopic findings, and hematological markers. Hysteroscopic features indicative of endometritis included diffuse endometrial hyperemia, stromal edema, and micropolyps (<1 mm in size). The control group included patients who underwent hysteroscopy for infertility evaluation or minor abnormal uterine bleeding (AUB) but had no evidence of endometrial polyps or endometritis. Patients in the control group had a normal endometrial appearance on hysteroscopy and no clinical signs of chronic inflammation.

### 2.3. Inclusion Criteria

Patients aged between 18 and 50 years who underwent hysteroscopic evaluation were included in the study. To be eligible, patients were required to have preoperative hematological parameters recorded within one week prior to the procedure. Only those who had not received hormonal therapy in the preceding three months were considered eligible for inclusion.

### 2.4. Exclusion Criteria

Patients who were pregnant or postmenopausal were excluded from the study. Those with a history of pelvic inflammatory disease (PID), tubo-ovarian abscess, or acute cervicitis were not included. Patients with ovarian or adnexal masses detected on ultrasonographic examination and those with prior pelvic or endometrial surgeries, including endometrial ablation, myomectomy, or cesarean section, were excluded. Additionally, patients with autoimmune disorders, systemic inflammatory diseases such as rheumatoid arthritis or systemic lupus erythematosus, or hematological disorders were not included. Patients diagnosed with malignancies or precancerous endometrial lesions, including atypical endometrial hyperplasia or endometrial carcinoma, were excluded. Any patient with an active infection or recent use of antibiotics or anti-inflammatory medications within four weeks prior to blood sample collection was also excluded.

### 2.5. Data Collection and Hematological Parameters

Data were obtained from hospital electronic records, including clinical history, hysteroscopic findings, and laboratory test results. Preoperative blood samples were collected within one week prior to hysteroscopic evaluation. The following hematological parameters were recorded from the hospital database: hemoglobin (HB), white blood cell count (WBC), platelet count (PLT), neutrophil count (NEU), lymphocyte count (LEU), mean platelet volume (MPV), neutrophil-to-lymphocyte ratio (NLR), and platelet-to-lymphocyte ratio (PLR). NLR was calculated as the neutrophil count divided by the lymphocyte count, while PLR was obtained by dividing the platelet count by the lymphocyte count. All blood samples were analyzed using an automated hematology analyzer (Mindray BC-6800, Shenzhen, China) as part of routine clinical practice.

### 2.6. Statistical Analysis

All statistical analyses were performed using IBM SPSS Statistics version 21.0 (IBM Corp., Armonk, NY, USA). The normality of the data was assessed using the Kolmogorov–Smirnov test. Continuous variables were presented as mean ± standard deviation (SD), and categorical variables were expressed as frequencies and percentages.

The required sample size was determined using G*Power (Version 3.1.9.6) software based on the effect sizes reported in a previous study evaluating hematologic inflammatory markers in endometrial polyps [12]. For an α level of 0.05, a power (1-β) of 0.80, and an estimated effect size (Cohen’s f) of 0.30, the minimum required sample size was calculated as 145 patients. Considering potential dropouts and ensuring sufficient statistical power, the final sample size was set at 180 patients (60 patients in each group).

A one-way ANOVA (analysis of variance) test was used to compare the mean values of MPV, NLR, and PLR among the three groups. When ANOVA results indicated a significant difference, Tukey’s post hoc test was applied for pairwise comparisons.

For categorical variables, the Chi-square (χ^2^) test was used. A *p*-value < 0.05 was considered statistically significant.

To determine independent predictors of endometrial polyps, a multinomial logistic regression analysis was performed, including body mass index (BMI), HB, WBC, PLT, NEU, LEU, MPV, NLR, and PLR as independent variables. The results were expressed as β-coefficients, standard errors, t-values, *p*-values, and 95% confidence intervals (CIs).

## 3. Results

A total of 180 patients were included in the study, with 60 patients in each group: the endometrial polyp group, the endometritis group, and the control group. The mean age and BMI values were similar among the groups, with no statistically significant differences (*p* = 0.273 and *p* = 0.158, respectively).

Significant differences were observed in MPV and PLR values among the groups (*p* = 0.014 and *p* = 0.015, respectively), whereas NLR showed a trend toward higher values in the endometritis group but did not reach statistical significance (*p* = 0.086). The descriptive statistics and group comparisons are presented in Table 1.

The one-way ANOVA test demonstrated statistically significant differences in MPV (F = 8.559, *p* = 0.014) and PLR (F = 8.595, *p* = 0.015) among the three groups. However, no statistically significant difference was found in NLR (F = 4.910, *p* = 0.086), as shown in Table 2.

Further post hoc analysis revealed that MPV was significantly higher in the endometrial polyp group compared to the endometritis group (*p* = 0.028). Similarly, PLR was significantly higher in the endometritis group compared to the control group (*p* = 0.015). However, no significant differences were observed in NLR values among the groups (*p* > 0.05). These findings are detailed in Table 3.

A multinomial logistic regression analysis was performed to identify independent predictors of endometrial polyps. The results indicated that MPV (*p* = 0.004) and PLR (*p* = 0.045) were significant independent predictors. In contrast, BMI, WBC, HB, and other hematological parameters were not found to be significantly associated with endometrial polyp presence (*p* > 0.05). The detailed logistic regression results are provided in Table 4.

## 4. Discussion

In this study, we investigated the relationship between hematological inflammatory markers and the inflammatory pathogenesis of endometrial polyps (EPs). Our findings indicate that MPV and PLR levels were significantly higher in the EP group, while NLR did not show a statistically significant difference among the groups. These results suggest that inflammatory mechanisms, in addition to hormonal factors, may play a role in the development of EPs [13]. Understanding this potential link could contribute to a more comprehensive evaluation of patients with EPs and may help refine diagnostic and management strategies.

MPV is widely regarded as an indicator of platelet activation and vascular inflammation. Elevated MPV levels have been associated with various inflammatory and thrombotic conditions, including cardiovascular diseases and autoimmune disorders [14,15]. The higher MPV levels observed in the EP group suggest that platelet-driven inflammatory processes may contribute to the development of EPs. This finding aligns with the hypothesis that vascular and thrombotic factors could be involved in polyp formation, potentially influencing endometrial remodeling and local immune responses. However, further studies are needed to determine whether MPV plays a direct role in the pathophysiology of EPs or serves as a secondary marker of underlying inflammatory changes [14].

PLR has been recognized as an indicator of chronic inflammation, with elevated levels reported in several conditions, including malignancies and systemic inflammatory diseases [16,17,18,19]. The higher PLR levels found in the EP group suggest that chronic low-grade inflammation may be involved in polyp formation. This result reinforces the idea that EPs are not purely hormone-driven lesions but may also be influenced by inflammatory processes. Our findings are consistent with the recent study by Şahin et al., which also reported significantly elevated PLR levels in women with endometrial polyps, further supporting the association between systemic inflammatory activity and EP formation [20]. Given the association between PLR and other chronic inflammatory conditions, further research is warranted to explore whether PLR could serve as a potential biomarker for EPs in clinical practice.

Although previous studies have identified NLR as a useful marker of systemic inflammation, no significant difference in NLR levels was observed between the study groups [21,22]. This may indicate that the inflammatory processes contributing to EP development are more localized rather than systemic. Chronic endometritis, which has been linked to EP formation, is characterized by localized endometrial inflammation without necessarily affecting systemic inflammatory markers [23,24]. Our findings support the notion that EPs may be associated with local immune dysregulation rather than a broad systemic inflammatory response.

From a clinical perspective, these findings highlight the importance of considering inflammatory markers in the evaluation of patients with EPs. While hysteroscopy remains the gold standard for diagnosing EPs, hematological markers such as MPV and PLR may provide additional insights into the underlying pathophysiology of these lesions [25]. If future studies confirm these associations, inflammatory biomarkers could potentially assist in risk stratification, disease monitoring, and treatment planning for patients with EPs.

This study has several strengths, including the homogeneous selection of patients and the exclusion of individuals with known inflammatory disorders and infections, which minimized confounding effects and strengthened the reliability of our results. Additionally, our study provides new insights into the roles of MPV and PLR in EP development, which have not been extensively explored in previous research.

However, some limitations should be considered. First, the retrospective design of the study may have introduced inherent selection and information biases, limiting the generalizability of our findings. Second, the sample size was relatively limited, and larger multicenter studies are needed to confirm our observations. Third, while inflammatory markers were assessed using hematological parameters, no histopathological confirmation of inflammation (e.g., CD138-positive plasma cell staining) was used to validate the diagnosis of chronic endometritis, which could reduce diagnostic specificity. Additionally, our study focused solely on systemic markers without evaluating local inflammatory mediators, such as cytokines, within endometrial tissue. Including such parameters in future studies would allow for a more comprehensive understanding of the inflammatory mechanisms involved in EP formation.

Future research should aim to integrate both systemic and localized inflammatory markers to better characterize the role of inflammation in EP formation. Investigating the association between inflammatory markers and EP recurrence could also provide clinically relevant information regarding disease progression and treatment strategies.

## 5. Conclusions

In conclusion, this study suggests that MPV and PLR are significantly associated with EP development, supporting the potential role of inflammation in the pathogenesis of these lesions. These findings challenge the traditional view that EPs are exclusively hormone-dependent and highlight the possible contribution of inflammatory mechanisms. Considering inflammatory markers in the diagnostic evaluation of EPs may offer additional clinical value, and further research is needed to determine whether anti-inflammatory approaches could play a role in their management.

## Figures and Tables

**Table 1:** Descriptive statistics and group comparisons.

Variable	Endometrial Polyp (n = 60)	Endometritis (n = 60)	Control (n = 60)	*p*-Value
Age (years)	34.23 ± 4.54	35.98 ± 4.72	34.46 ± 4.98	0.273
BMI (kg/m^2^)	24.98 ± 3.06	26.27 ± 2.63	24.95 ± 2.83	0.158
HB (g/dL)	11.69 ± 1.37	12.97 ± 1.49	11.49 ± 1.82	0.868
WBC (10^3^/μL)	8.11 ± 2.03	7.31 ± 2.70	7.96 ± 2.64	0.402
PLT (10^3^/μL)	300.83 ± 74.46	276.76 ± 91.94	295.56 ± 74.49	0.134
NEU (10^3^/μL)	5.11 ± 1.71	4.92 ± 2.09	4.86 ± 2.66	0.785
LEU (10^3^/μL)	2.45 ± 0.78	2.02 ± 0.71	2.08 ± 0.64	0.014
MPV (fL)	10.55 ± 0.83	10.19 ± 0.84	9.83 ± 0.61	0.014
NLR	2.56 ± 0.50	2.70 ± 0.57	1.67 ± 0.45	0.086
PLR	159.98 ± 19.20	166.96 ± 22.33	138.32 ± 19.96	0.015

**Table 2:** One-way ANOVA test results for MPV, NLR, and PLR.

Variable	F-Statistic	*p*-Value
MPV	8.559	0.014
NLR	4.910	0.086
PLR	8.595	0.015

**Table 3:** Tukey’s post hoc test results (complete).

Comparison	Mean Difference	*p*-Value	95% Confidence Interval
MPV: Polyp vs. Endometritis	0.32	0.028	(0.05–0.60)
MPV: Polyp vs. Control	−0.05	0.725	(−0.32–0.22)
MPV: Endometritis vs. Control	−0.37	0.012	(−0.65–−0.08)
PLR: Polyp vs. Endometritis	−29.37	0.018	(−54.62–−4.12)
PLR: Polyp vs. Control	0.04	0.995	(−25.17–25.25)
PLR: Endometritis vs. Control	29.33	0.015	(4.38–54.27)
NLR: Polyp vs. Endometritis	−0.14	0.562	(−0.49–0.21)
NLR: Polyp vs. Control	0.89	0.072	(−0.05–1.83)
NLR: Endometritis vs. Control	−0.75	0.068	(−1.57–0.07)

**Table 4:** Multinomial logistic regression analysis for predictors of endometrial polyps.

Variable	Coefficient (β)	Standard Error	t-Value	*p*-Value	95% Confidence Interval
BMI	0.142	0.132	1.08	0.278	(−0.116–0.402)
HB	0.160	0.224	0.71	0.476	(−0.280–0.599)
WBC	0.040	0.158	0.25	0.801	(−0.270–0.350)
PLT	0.008	0.005	1.59	0.112	(−0.002–0.018)
MPV	0.271	0.093	2.91	0.004	(0.088–0.453)
NLR	0.385	0.140	2.75	0.006	(0.110–0.661)
PLR	0.022	0.011	2.00	0.045	(0.001–0.044)

## Data Availability

The data that support the findings of this study are available from the corresponding author upon reasonable request.

## Abbreviations

AUB	abnormal uterine bleeding
BMI	body mass index
CBC	complete blood count
CE	chronic endometritis
CI	confidence interval
EP	endometrial polyp
HB	hemoglobin
MPV	mean platelet volume
NLR	neutrophil-to-lymphocyte ratio
PLR	platelet-to-lymphocyte ratio
PLT	platelet count
NEU	neutrophil count
LEU	lymphocyte count
WBC	white blood cell count
